# Autologous Concentrated Bone Marrow Grafting for the Treatment of Osteonecrosis of the Humeral Head: A Report of Five Shoulders in Four Cases

**DOI:** 10.1155/2017/4898057

**Published:** 2017-06-20

**Authors:** Takeshi Makihara, Tomokazu Yoshioka, Hisashi Sugaya, Masashi Yamazaki, Hajime Mishima

**Affiliations:** ^1^Department of Orthopaedics Surgery, Faculty of Medicine, University of Tsukuba, 1-1-1 Tennodai, Tsukuba, Ibaraki 305-8575, Japan; ^2^Division of Regenerative Medicine for Musculoskeletal System, University of Tsukuba, 1-1-1 Tennodai, Tsukuba, Ibaraki 305-8575, Japan

## Abstract

Five shoulders in four patients affected by advanced osteonecrosis of the humeral head were treated with autologous concentrated bone marrow grafting. Bone marrow sample was aspirated from the iliac crests, concentrated by a centrifugation technique, and injected into the necrotic site. The shoulders were evaluated radiologically with X-ray scoring and clinically with measurement of range of motion and pain score (visual analogue scale, VAS). The mean follow-up period was 49.4 (range, 24–73) months. The concentration ratio of nucleated cells was calculated and the number of transplanted mesenchymal stem cells (MSC) was estimated by a colony-forming assay. All four shoulders with stage 3 disease achieved joint sparing. One shoulder with stage 4 disease required replacement surgery. Clinical evaluation of the spared joints showed improvement in range of motion in two cases and deterioration in two cases. VAS scores were 0 after surgery in three cases. The mean concentration ratio was 2.73, and the mean number of transplanted MSC was 1125. The outcomes of autologous concentrated bone marrow grafting for advanced osteonecrosis of the humeral head were varied. Further research is needed to determine the effectiveness and the indications of the present surgery.

## 1. Introduction

The humeral head is the most frequent site for nontraumatic osteonecrosis, followed by the femoral head [1], and humeral head osteonecrosis is present in 13–25% of cases of femoral head necrosis [2, 3]. Osteonecrosis may also develop following trauma and is a complication of 26–75% of Neer classification type 3 and 4 proximal humerus fractures [4–6]. Patients are often asymptomatic in the initial stages before developing pain-related collapse or limited range of motion that affects activities of daily living [7–13]. The consequent osteoarthritis of the shoulder is often treated with hemiarthroplasty or with total arthroplasty; however, these procedures have issues regarding durability and risk of complications such as infection [14–17]. Because nontraumatic osteonecrosis is frequently caused by steroid administration and commonly affects patients aged between 30 and 40 years with high activity levels [18], joint-sparing treatment of the affected cases is important.

Reported joint-sparing approaches for osteonecrosis of the humeral head include conservative follow-up [19–21], bone grafting [22, 23], and core decompression [14, 18]. Arthroplasty is required in 26–54% of cases managed by conservative follow-up [19–21]. A small number of autogenous bone grafting studies have been reported, including the strut bone graft [22] and the vascularized scapular bone graft [23]; however, these procedures are complex. Core decompression is a straightforward procedure achieving favorable clinical results in 91–100% of cases identified prior to collapse [24, 25]. Therefore, the procedure is considered a useful joint-sparing treatment if performed at an early stage of the disease; however, positive outcome decreases to 57% in cases following collapse. The most effective joint-sparing treatment of the humeral head in cases following collapse remains an unresolved issue.

Core decompression can be applied to osteonecrosis of the femoral head with consistent results [26]. In addition, methods for the transplantation of mesenchymal stem cells have been reported with superior outcomes [27]. The University of Tsukuba currently performs autologous concentrated bone marrow grafting for osteonecrosis of the femoral head [28]. A bone tunnel is created through the lateral cortex to reach the necrotic bone before transplantation of centrifugally concentrated bone marrow aspirate. Multipotent mesenchymal stem cells are found in bone marrow aspirate [29] and can be concentrated approximately 5-fold by centrifugation [30]. The osteogenic ability of bone marrow aspirate has been demonstrated in experiments using animal osteonecrosis models [31], and mesenchymal stem cells within bone marrow aspirate have been shown to directly differentiate into new bone [32]. In addition to direct communication with the normal environment of the surrounding tissues enabled by the creation of the bone tunnel, it is assumed that a mechanism exists where bone formation by transplanted concentrated bone marrow aspirate suppresses osteonecrosis progression.

Although the humeral head has a different load environment and range of motion when compared to the femoral head, subchondral osteonecrosis develops in both bones. The pathophysiology of both conditions is believed to be similar; therefore, we propose the hypothesis that transplantation of concentrated bone marrow aspirate in addition to the creation of a bone tunnel would be effective. We conducted a retrospective study of five autologous concentrated bone marrow grafting procedures in four advanced cases of humeral head osteonecrosis.

## 2. Methods

A total of 11 shoulders affected by osteonecrosis of the humeral head were identified in eight patients between 2008 and 2013. Surgery was performed on seven shoulders in six patients without improvement following conservative treatment for ≥6 months. We excluded two shoulders of two patients with complicated posttraumatic pseudarthrosis. A total of five shoulders in four patients were included in this study. Patient clinical characteristics are presented in Table 1. The mean age was 48 (range, 38–63) years; there was one male patient (two procedures) and three female patients (one procedure each). Three patients (four procedures) had a history of steroid use and complicated osteonecrosis of the femoral head. One case was traumatic and likely developed following open reduction and internal fixation (ORIF) using screws. The average postoperative follow-up period was 49.4 (24–73) months.

## 3. Surgery

The collection of bone marrow aspirate, centrifugation, and transplantation procedures were performed in accordance with a previous report by Yoshioka et al. [28]; the technique was performed in osteonecrosis cases involving the femoral head. Bone marrow aspirate was harvested from the ilium under general anesthesia using a syringe containing acid citrate dextrose (ACD) with a bone marrow biopsy needle (Baxter, United States) and collected in a blood bag (Terumo, Japan). The red blood cell layer separated following centrifugation at 1200 ×g for 10 min was manually removed. Centrifugation was then performed at 3870 ×g for 7 min and separated blood plasma was removed. The remaining buffy coats were moved to a syringe. Patients were moved to a beach chair following blood collection to alter body position before confirming the feasibility of fluoroscopy from two directions. A 2.4 mm diameter guide pin was inserted percutaneously into the osteonecrosis site; then a small incision was made at the pin insertion site. Drilling was performed with a 4.8 mm diameter drill using the guide pin as the guide. Placement in the osteonecrotic site was confirmed by fluoroscopy and the loss of resistance derived from the peripheral sclerosis zone of the osteonecrotic site. Autologous concentrated bone marrow was then grafted using a 3.8 mm diameter cylindrical shaped rod. No specific instructions were given to patients postoperatively other than to avoid carrying loads.

## 4. Evaluation

Radiological and clinical functional evaluations were performed preoperatively and at the final follow-up examination. Plain radiography was used to radiologically classify diseases using the Cruess classification [19]. Stage 1 describes no obvious change on radiographs with the diagnosis only possible by magnetic resonance imaging (MRI), bone scintigraphy, or biopsy; stage 2 describes cases with localized bone sclerosis or bone translucency on simple radiograph without major changes in morphology; stage 3 describes cases in which a fracture line of the subchondral bone, known as a crescent sign, is observed with development of mild collapse; stage 4 describes cases with the obvious development of collapse; and stage 5 describes cases with osteoarthritic changes.

The evaluation of clinical function included the range of motion and degree of pain. Shoulder joint flexion and the abduction angle were measured to assess range of motion, and the visual analogue scale (VAS) was used for pain evaluation. The case that resulted in joint replacement surgery was excluded from final clinical functional evaluation.

## 5. Blood Analysis

We performed blood analysis according to the methods reported by Sakai et al. [30]. The total amounts of bone marrow aspirate collected and transplanted were recorded. Concentration ratios were calculated from the nucleated cell count before and after concentration. The concentration of mesenchymal stem cells in the transplanted material was estimated via a fibroblastic colony-forming-unit (CFU-F) assay, and the total number of transplanted mesenchymal stem cells was calculated by multiplying this value by the amount of bone marrow transplanted.

## 6. Results

The list of results is shown in Table 2. No complications due to the surgery were observed.

Preoperatively, four shoulders had stage 3 disease and one shoulder had stage 4 disease. At the final follow-up examination, three shoulders had stage 3 disease, which had not progressed since surgery; however, one shoulder had progressed from stage 3 to stage 4 disease, and one shoulder with preoperative stage 4 disease required replacement surgery 20 months after the original surgery. Clinically, range of motion improved to full range in one stage 3 shoulder and in one stage 4 shoulder and deteriorated in two stage 3 shoulders. The VAS score was 0 in two stage 3 shoulders and one stage 4 shoulder.

The average amount of bone marrow aspirate collected was 230 mL (120–400 mL). The average amount transplanted was 20.8 mL (8–32 mL). Concentration rates were calculated, except in Case 1, because blood analysis data was lost. The mean concentration ratio was 2.73 (2.45–3.06). CFU-F data was lost for Case 2 because of infection of the concentrated bone marrow aspirate culture. The transplanted MSC count was calculated for all cases except Case 2; the mean number was 1125 (283–2462).

*Case Presentation #1 (A 38-Year-Old Man)*. The patient had been taking steroids for Harada's disease. The patient reported pain in both shoulders for 3 years following initiation of steroid treatment and was diagnosed with osteonecrosis of the humeral head. Pain remained following conservative follow-up, and surgery was performed for the bilateral shoulders 2 years after the initial diagnosis.

**Figure 1:**
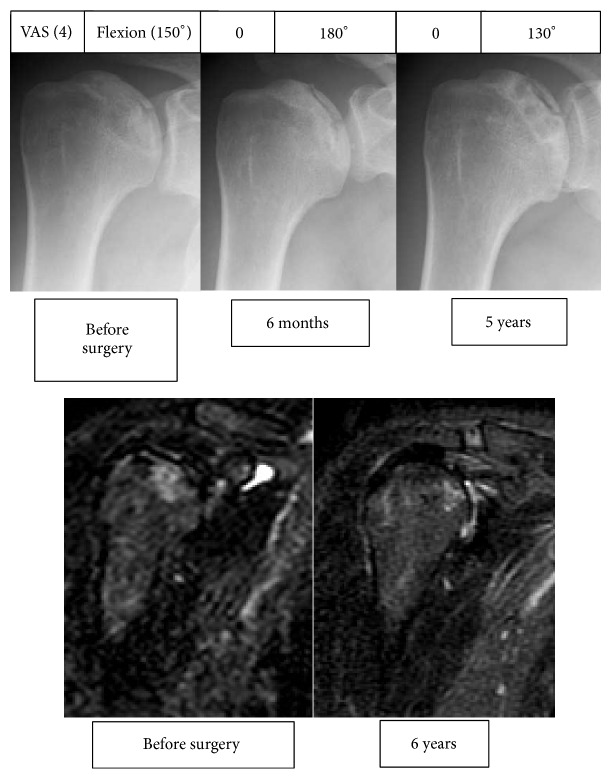
Radiographic and magnetic resonance images of the right shoulder in Case 1. The box on top of the radiographic images shows the visual analogue scale on the left and the range of flexion on the right.

The patient had a preoperative flexion range of 150°, abduction range of 80°, and VAS score of 40 when at rest in both shoulders. The advanced stage classification was that of stage 3 disease. Pain resolved 6 months after surgery in the right shoulder but remained in the left shoulder. At postoperative year 5, the flexion range was 130°, abduction range was 100°, and VAS score was 0 in the right shoulder, while the flexion range was 120°, abduction range was 100°, and VAS score was 20 in the left shoulder. No disease progression was observed in either shoulder according to the stage classification, and the score remained at stage 3 at postoperative year 5. Bone marrow edema in the right shoulder was found to have decreased on MRI (Figure 1).

*Case Presentation #2 (A 48-Year-Old Woman)*. The patient sustained a fracture of the proximal left humerus due to a fall during skiing. ORIF was performed for a valgus impacted 4-part fracture. The impacted humeral head was reduced by elevation, and bone substitute was transplanted into the area of bone loss. Fixation was performed with cannulated cancellous screws (CCS). Bone union was achieved, but osteonecrosis of the humeral head was detected 18 months postoperatively.

**Figure 2:**
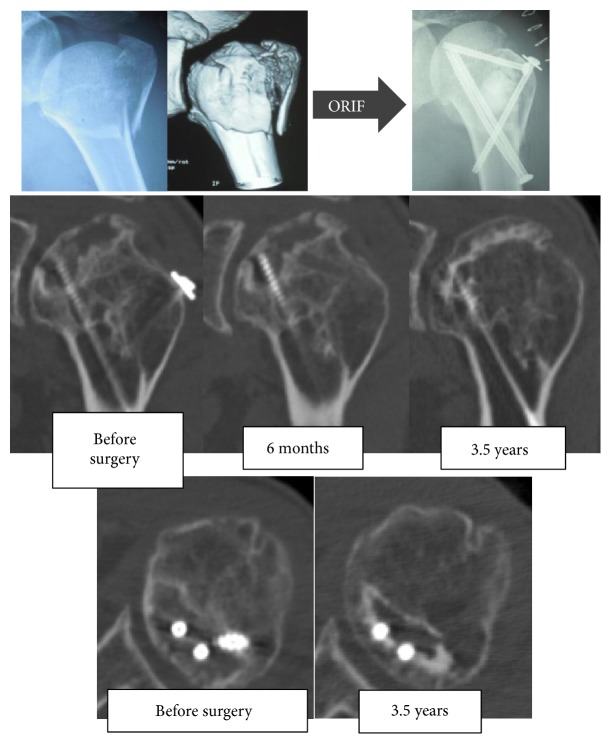
X-ray and computed tomography images of Case 2.

Although the patient did not feel any pain, the flexion angle was limited to 130°, and the abduction angle was limited to 85°. Bone loss in the subchondral bone was observed on preoperative computed tomography, but bone loss was decreased at 6 months and 3.5 years after surgery with improved articular congruence (Figure 2). No pain or limited range of motion was observed at 3.5 years postoperatively.

*Case Presentation #3 (A 63-Year-Old Woman)*. The patient had been taking steroids for dermatomyositis. The patient reported pain in the right shoulder for 7 years following the initiation of steroid treatment and was diagnosed with osteonecrosis of the humeral head. Pain remained following conservative follow-up, and surgery was performed for the right shoulder 4 months after the initial diagnosis.

**Figure 3:**
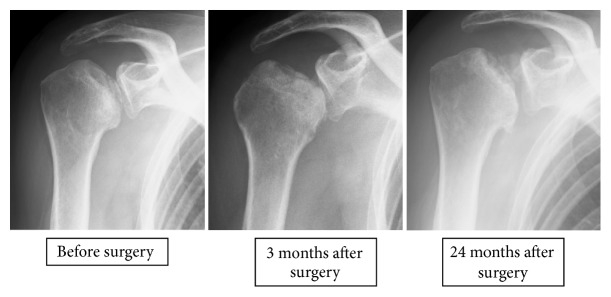
X-ray images of Case 3.

The patient had a preoperative flexion range of 140°, abduction range of 170°, and VAS score of 52. The advanced stage classification was that of stage 3 disease. Pain resolved 2 months after surgery and showed no deterioration during the 2-year observation period. At postoperative year 2, the flexion range was 180°, abduction range was 180°, and VAS score was 0, in spite of radiological progression from stage 3 to stage 4 (Figure 3).

*Case Presentation #4 (A 43-Year-Old Woman)*. The patient had been taking steroids for mixed connective tissue disease. The patient reported pain in the left shoulders for 2 years following initiation of steroid treatment and was diagnosed with stage 3 osteonecrosis of the humeral head. During conservative follow-up, the humeral head showed collapse, resulting in radiological progression to stage 4. Surgery was performed for the left shoulder 6 months after the initial diagnosis.

**Figure 4:**
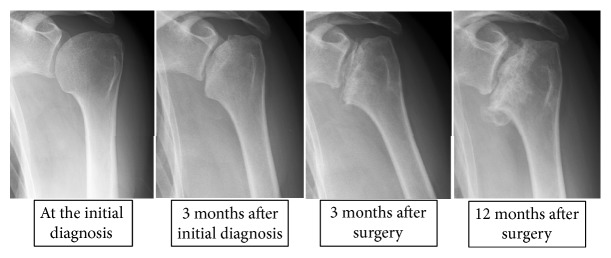
X-ray images of Case 4.

The patient had a preoperative flexion range of 100°, abduction range of 60°, and VAS score of 40. Pain reduced after surgery for 10 months; however, pain then recurred with radiological progression to stage 5 (Figure 4) and the patient required replacement surgery 20 months after the original surgery.

## 7. Discussion

In the present study, we report five shoulders affected by advanced osteonecrosis after collapse treated with autologous concentrated bone marrow grafting. Four shoulders with preoperative stage 3 disease achieved joint sparing with favorable clinical results despite radiological progression in one case. One shoulder with preoperative stage 4 disease failed joint sparing, resulting in replacement surgery.

The natural course of advanced osteonecrosis of the humeral head has been reportedly dismal. Hattrup and Cofield reported the conservative follow-up of 200 total cases with osteonecrosis of the humeral head, including 94 cases with stage 3 or 4 disease; their results demonstrated that 37.9% (11/29) of stage 3 patients and 66.1% (43/65) of stage 4 patients required replacement surgery [20]. Poignard et al. reported the natural progression of symptomatic humeral head osteonecrosis in adults with sickle cell disease, demonstrating that 70.8% (17/24) of cases with advanced stage disease required surgical treatment [13]. These studies are consistent with the patients in our institution, where 7 out of 11 patients with humeral head osteonecrosis required surgical treatment.

The present cases required surgical treatment due to persistent symptoms following conservative follow-up consisting of rest, medication, and rehabilitation. The patients wished to have additional treatment. However, these patients were young, with a mean age of 48 years; therefore, joint replacement would likely require future revision surgery, and a joint-sparing approach was required.

The established joint-sparing method for osteonecrosis of the humeral head is core decompression [25, 33]. Mont et al. performed core decompression in 20 cases with osteonecrosis of the humeral head using a 5 mm drill [33], and further cases were reported by LaPorte et al., who examined the outcome of 67 shoulder procedures in 46 patients over an average follow-up duration of 10 (2–20) years [25]. According to this report, 91% (30/33) of early stage cases obtained favorable outcome; however, 43% (13/30) of the cases in advanced stage with collapse showed unfavorable results. These results were not likely sufficient to convince the patients presented here. Vascularized bone graft could be indicated for their condition; however, surgery represents a complex option, being highly invasive and requiring microsurgery techniques. We expect our technique, which has been used for osteonecrosis of the femoral head, to be another treatment option for osteonecrosis of the humeral head, representing a more simple and less invasive method. Therefore, we decided to perform autologous concentrated bone marrow grafting. These were our first attempts to apply our original method to the humeral head, and, to the best of our literature research, this is the first report of the treatment with combined core decompression for osteonecrosis of the humeral head.

Our results demonstrated conservation of the humeral head in all cases of preoperative stage 3 disease with alleviation of pain. Range of motion improved in two cases and deteriorated in two cases. These results suggest potential efficacy for the treatment of stage 3 advanced humeral head osteonecrosis; however, clinical outcomes were varied in this case series. An apparent decrease of the bone defect observed in Case 2 indicated that the transplantation of concentrated bone marrow aspirate and core decompression could prevent advancing collapse through early bone formation. This was the only case of posttraumatic osteonecrosis showing acceptable radiological and clinical outcome. Possible reasons of the improved outcome were her better bone quality and activity level (the patient is a licensed skier) compared to other patients with connective tissue disease treated using corticosteroids; however, the precise etiology is unclear. Case 3 progressed from stage 3 to stage 4 disease on imaging. Case 3 was of older age and had been treated using corticosteroids, which could result in osteoporosis. Bone quality could also be a factor influencing the radiographic result; however, she retains satisfactory clinical function at present. Function and pain of the shoulder are not always related to radiological findings [34], an important fact when planning the treatment strategy for osteonecrosis of the humeral head. In the same way, range of motion deteriorated in spite of good radiological result in Case 1. Further research with a larger number of cases is needed to determine the effectiveness and the indications of the present surgery.

Case 4 showed preoperative stage 4 disease and alleviation of pain postoperatively; however, shoulder pain relapsed and the humeral head could not be saved. Although autologous concentrated bone marrow grafting might contribute to pain relief in the short term, early detection and treatment are more important.

This study has a number of limitations. Because we used a 4.8 mm drill and had a different follow-up duration than previous studies, our results are not directly comparable. Pain alleviation and bone formation may be obtained with core decompression alone; therefore, we are unable to fully assess the effectiveness of the method used in this study because of the small number of included cases. A well-designed randomized study is instead required.

In this case series, the concentration ratio of transplanted bone marrow aspirate was 2.73, which was lower than that in previous reports. In addition, the number of transplanted mesenchymal stem cells, estimated by CFU-F, varied between 283 and 2462. This variation may be because of interpersonal differences in the amount of bone marrow aspirate collected using this technique or the manual operation of centrifugation.

## 8. Conclusion

Autologous concentrated bone marrow grafting was performed in four shoulders of three patients with stage 3 osteonecrosis of the humeral head with variable clinical outcomes. One patient with stage 4 osteonecrosis of the humeral head required replacement surgery.

## Figures and Tables

**Table 1:** Clinical profiles of patients.

Number	Sex	Age (years)	Follow-up period (months)	Operated side	Etiology
1	M	38	73	Right	Corticosteroid
			73	Left	Corticosteroid
2	F	48	53	Left	Trauma
3	F	63	24	Right	Corticosteroid
4	F	43	24	Left	Corticosteroid

**Table 2:** Clinical results and blood analysis.

Before surgery				After surgery				Concentration ratio	Transplanted MSCs
Stage	VAS (mm)	Range of motion		Stage	VAS (mm)	Range of motion			
		Flexion	Abduction			Flexion	Abduction		
3	40	150	80	3	0	130	100	—	2462
3	40	150	80	3	20	120	100	—	1407
3	0	130	85	3	0	180	180	2.45	—
3	52	140	170	4	0	180	180	3.06	350
4	40	100	60	5^*∗*^	—	—	—	2.69	283

VAS: visual analogue scale, MSC: mesenchymal stem cell. ^*∗*^Patient 4 required replacement surgery 20 months after the surgery.
